# The Human Functional Brain Network Demonstrates Structural and Dynamical Resilience to Targeted Attack

**DOI:** 10.1371/journal.pcbi.1002885

**Published:** 2013-01-24

**Authors:** Karen E. Joyce, Satoru Hayasaka, Paul J. Laurienti

**Affiliations:** 1 School of Biomedical Engineering and Sciences, Wake Forest University School of Medicine, Winston-Salem, North Carolina, United States of America; 2 Department of Biostatistical Sciences, Wake Forest University School of Medicine, Winston-Salem, North Carolina, United States of America; 3 Department of Radiology, Wake Forest University School of Medicine, Winston-Salem, North Carolina, United States of America; Centre National de la Recherche Scientifique, France

## Abstract

In recent years, the field of network science has enabled researchers to represent the highly complex interactions in the brain in an approachable yet quantitative manner. One exciting finding since the advent of brain network research was that the brain network can withstand extensive damage, even to highly connected regions. However, these highly connected nodes may not be the most critical regions of the brain network, and it is unclear how the network dynamics are impacted by removal of these key nodes. This work seeks to further investigate the resilience of the human functional brain network. Network attack experiments were conducted on voxel-wise functional brain networks and region-of-interest (ROI) networks of 5 healthy volunteers. Networks were attacked at key nodes using several criteria for assessing node importance, and the impact on network structure and dynamics was evaluated. The findings presented here echo previous findings that the functional human brain network is highly resilient to targeted attacks, both in terms of network structure and dynamics.

## Author Summary

Why can the brain endure numerous micro-strokes with seemingly no detrimental impact, until one cataclysmal stroke hinders the ability to perform essential functions such as speech and mobility? Perhaps various small regions or foci of the brain are highly important to information transfer, and the loss of such highly central foci would be severely injurious to brain function. Identification of such foci, via modeling of the functional brain using network theory, could lead to important advances with regard to brain disease and stroke. In this work, we utilized functional brain networks constructed from human volunteers to study how removing particular regions of the brain impacts brain network structure and information transfer properties. We sought to determine whether a particular measure of region importance may be able to identify highly critical regions, and whether targeting highly critical regions would have a more detrimental impact than removing regions at random. We found that, while in general targeted removal has a larger impact on network structure and dynamics, the human brain network is comparatively resilient against both targeted and random removal.

## Introduction

Complex systems may be represented as networks by modeling the system components as nodes and the interactions between components as links, and graph theory methods and dynamical simulations may then be applied to these networks in order to understand their structure and dynamics. The human brain is an example of such a system that can be described as a network. The functional relationships between brain regions, typically measured using imaging techniques such as functional magnetic resonance imaging (fMRI), can be described as a brain network; in particular, nodes represent various brain regions and edges represent strong coherence among the nodes. For a review of the construction and analysis of functional brain networks, we refer the reader to [1] and [2].

An exciting finding since the advent of brain network research was that the functional brain network can withstand extensive damage, even to highly connected regions [3]. In this prior work, regions of the brain network were systematically attacked based on their degree, the number of links to which each region was functionally connected. Regions having the highest degree were eliminated and the associated changes on network topology were evaluated. Then the next highest degree nodes were identified and eliminated and the changes in the network topology were recorded. This process was repeated until all nodes of the network had been removed. This type of systematic removal is referred to as targeted attack, where the most critical hubs are targeted for removal. Additionally, the effect of random failure was studied by selecting nodes for removal with uniform probability.

Achard et al. compared the resilience of brain networks to that of two null models, random networks and scale-free networks, since the level of robustness of these networks had been studied previously [4]. Random networks, where the majority of nodes have a similar number of connections (or degrees), proved to be highly resilient to both targeted attack and random failure. Scale-free networks, on the other hand, fragmented rapidly. This may be because a scale-free network is highly vulnerable at a very small number of high-degree nodes, or mega-hubs, which mediate connections among low degree nodes constituting the majority of the network [4]. Functional brain networks, while not as resilient as random networks, were shown to be far more robust than scale-free networks. It is well known that brain networks have characteristics of small-world architecture, that is a combination of high clustering for local specialization and low path length to enable distributed processing [5], [6], [7]. Achard et al. proposed that the resilience of the brain network was due to this small-world architecture. Furthermore, Achard et al. observed that the functional brain network degree distribution followed an exponentially truncated power law, meaning that there are fewer mega-hubs and a greater number of mid-degree nodes than would be expected in a scale-free distribution. This exponentially truncated power law distribution also likely contributed to the resilience against targeted attacks of hubs.

However, it is possible that the highest degree nodes are not the most critical nodes of the brain network [8]. There are many measures of node importance, or centrality. Each centrality metric has a different consideration for the topological properties that make a node central, and therefore different centrality metrics may be more appropriate for different networks and their specific information flow processes [9]. Furthermore, it is unclear how the removal of these nodes may impact network dynamics in addition to topology.

Alstott et al. have taken significant strides towards studying how failure of nodes in the brain network may impact network dynamics [10]. Their study involved simulating neural dynamics on structural brain networks constructed from diffusion spectrum imaging data. These simulated neural dynamics were used to create functional connectivity networks. Network nodes were eliminated based on degree, strength (weighted degree), and betweenness centrality to study the effect on topology. The impact was evaluated by calculating changes in global efficiency and the size of the largest connected component. In dynamical simulations, lesions were simulated by targeting groups of nodes centered on anatomical locations. The impact of a particular lesion was evaluated by simulating neural dynamics on the lesioned networks, and noting changes in the resulting functional networks. They found that betweenness centrality had a considerable impact on network topology, and that the effect on network dynamics is highly dependent on the anatomical location of the lesion.

Another study evaluated the effect of brain lesions due to stroke, traumatic brain injury, and brain tumors on functional brain network structure [11]. Specifically, Gratton et al. were concerned with the impact of lesions on brain network community structure, the topological property where network nodes tend to associate into well connected groups. Images from healthy participants and patients with lesions were used to create networks with approximately 90 nodes, in which corresponding nodes in each population were mapped to the same anatomical space. Each network was partitioned into modules (communities) using Newman's modularity [12]. Each node was evaluated for its within-module degree, or the number of links connecting nodes in the same module, as well as its participation coefficient, a summary metric of how diversely the node is connected to multiple modules. Gratton et al. discovered that the networks of lesioned patients had lower modularity scores when the lesions were in areas that exhibited higher participation coefficients in normal subjects. There was no statistical relationship between the within-module degree of lesioned nodes and the effect on modularity. They concluded that damage to brain regions linking multiple modules leads to a reorganization of the network that is detrimental to the entire network topology.

A large body of previous work on dynamics in complex networks has been focused on artificial networks. Watts studied global cascades in random networks due to small perturbations in the signals embedded in the network [13]. In these networks, each node has a state (either 1 or 0), and it may choose to change its state based on the states of its neighboring nodes according to a threshold rule. A cascade occurs when a few nodes switch states, causing a large scale propagation of state-switching throughout a large portion of the network. Watts found that as the distribution of threshold values for state-switching was made to be more heterogenous, the system became more prone to producing large cascades. In a similar experiment studying cascades in coupled map lattices, Wang and Xu noted that the size of the cascade is highly dependent on the network structure [14]. They showed that coupled map lattices with small-world architecture or scale-free degree distributions are much more likely to exhibit large cascades due to local shocks than globally coupled (fully connected) lattices. Rubinov et al. designed a neurobiologically relevant dynamic model consisting of a computerized network of spiking neurons [15]. They investigated the topological factors necessary for the emergence of self-organized criticality, marked by system dynamics that are self-similar on multiple spatial and temporal scales. They found that the presence of community structure (groups of nodes that are tightly interconnected), low wiring cost (an estimation of the average distance each wire traverses across the network), and synaptic plasticity were all necessary components for producing self-organized criticality. Tanaka et al. studied targeted attack on networks of coupled oscillators [16]. They discovered that the removal of low degree nodes has a large effect on the dynamics of these networks while the removal of high degree nodes does not. They speculate that this is due to the fact that low degree nodes do not interact with a large number of other nodes and therefore have the ability to sustain high levels of activity. As such, the removal of low degree nodes has the potential to alter the overall activity in the system to a great extent.

Despite all of the important work on the topological resilience of functional brain networks to targeted attack, and impact on the dynamics of artificial networks, it is still not clear how targeted attacks impact the dynamics in functional brain networks. In this work, we sought to expand our understanding of the resilience of the human functional brain network, both in terms of topology and dynamics. We conducted targeted attack experiments on voxel-based functional brain networks and region-of-interest (ROI) networks of 5 healthy volunteers. Networks were selectively attacked using several node centrality metrics to determine which centrality metric best identifies critical nodes. We measured the resulting impact on network topology using three criteria, and utilized two frameworks for assessing the dynamical impact.

## Materials and Methods

All experiments were conducted in accordance with the ethical standards of the Wake Forest University institutional review board and with the Helsinki Declaration of 1975. Functional brain networks of 5 healthy volunteers were constructed according to [8]. For each subject, 120 fMRI full-brain volumes were acquired over approximately 5 minutes. Images were corrected for motion, normalized to the MNI (Montreal Neurological Institute) space, and re-sliced to 4×4×5 mm voxel size using SPM99 (Wellcome Trust Centre for Neuroimaging, Longdon, UK). From these volumes, one time series was extracted for each of the 15,996 voxels encompassing all of the gray matter of the cerebrum. Images were corrected for physiological noise by band-pass filtering to eliminate signal outside of the range of 0.009–0.08 Hz [17], [18], and mean time courses from the entire brain, the deep white matter, and the ventricles were regressed from the filtered time series. In the past, the practice of global mean regression has been under scrutiny due to the propensity to produce artificial deactivations, particularly in the white matter and cerebrospinal fluid (CSF) [19]. It is important to note, however, that failure to regress the mean signal will prevent detection of true deactivations that are known to occur in the brain. Additionally, the regions that are highly sensitive to these artifacts (white matter and CSF) are not considered in the present work. A full discussion on this topic can be found in [20].

**Figure 1:**
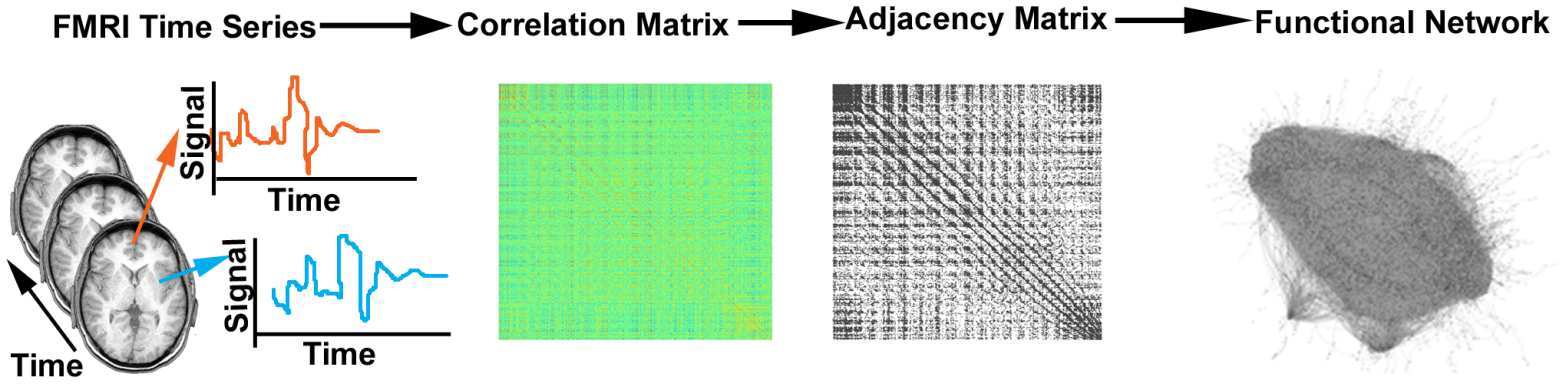
Generating a functional brain network. Functional magnetic resonance imaging (fMRI) data are collected from a subject, yielding a time series for each gray matter voxel in the cerebrum. The correlation values between each voxel are calculated to produce a correlation matrix. A threshold is applied to the correlation matrix to create an adjacency matrix, where all values surviving the threshold are set to 1. This adjacency matrix defines the links present in the functional brain network.

The time series in each voxel was correlated with every other voxel using the Pearson's correlation coefficient. These correlation values were then represented in a correlation matrix summarizing the functional relationships between every pair of voxels. A threshold was applied to the correlation matrix, above which voxel pairs were said to be connected. This resulted in a binary adjacency matrix where 1 indicated the presence of a link and 0 indicated the absence. The threshold was defined such that the relationship between the number of nodes *N* and average number of connections between nodes *k* was consistent across subjects. Specifically, the ratio of log(*N*) to log(*k*) was the same across subjects [21]. This threshold resulted in a link density of approximately 0.0015, where density is the ratio of the number of links present in the network to the number of possible links. This density is consistent with the size-density relationship of many self-organized networks described in [22]. Moreover, links defined by this threshold represented correlations that are approximately 3 standard deviations above the mean. **Figure 1** depicts the process of generating the functional brain networks.

Each functional brain network was selectively attacked at the nodes with the highest centrality. In particular, the top 5% highest centrality nodes were removed from the network, along with any links directly connected to those regions. After the removal of the nodes, the respective centrality measure was recalculated and another set of top 5% nodes were identified. This process was repeated until all nodes in the network were removed. Four centrality metrics were utilized, namely, degree centrality, leverage centrality, eigenvector centrality, and betweenness centrality. Degree centrality defines highly central nodes to be those having a high number of links connected to that node. Leverage centrality relates the degree of a node to that of its immediate neighbors. In particular, nodes with higher degrees than their neighbors are considered highly central to their local neighborhood [8]. Eigenvector centrality evaluates centrality based on the centrality of immediately connected neighbors, and therefore a node connected to nodes with high degree is highly central by association [23]. Betweenness centrality defines the importance of a node by the number of shortest paths between pairs of nodes on which the node lies. In this way, high betweenness nodes facilitate the exchange of information along the most efficient trajectories [24]. Formulations for these metrics are provided in [8]. In addition to targeted attacks, we also conducted random attacks by iteratively removing 5% of nodes randomly at each step.

After attacking the networks, changes in the network structure were evaluated by assessing three network characteristics: local efficiency (*E_loc_*), global efficiency (*E_glob_*), and the size of the giant component (*S*). Local and global efficiency are used to infer the efficacy of information exchange through a network by studying its topology [25]. Local efficiency quantifies the extent to which nodes communicate with immediate neighbors and can be thought of as an indication of regional specificity. Global efficiency quantifies the extent to which nodes communicate with distant nodes, and indicates the efficacy of information exchange throughout the entire network. As nodes are removed, the network may fragment into isolated subgraphs. The size of the giant component is defined to be the largest connected subgraph, and may be used to indicate the extent of fragmentation.

The impact on dynamics was evaluated using two models. The first is an equation-based spreading activation model described in [26]. This model injects signal into a network, and allows the signal to spread through links and decay according to model parameters. The equation governing the spread of activation is given in **Equation 1** below.
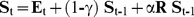
(1)


If N is the number of nodes in the network, **S_t_** is an N×1 vector describing the signal at time t, **E_t_** is an N×1 vector containing the external signal injected at time t, γ is the relaxation rate of the signal (0≤γ≤1), α is the relative amount of activity that flows from a node to its neighbors per unit time (α>0), and **R** is the N×N connectivity matrix. **R** was constructed by eliminating all negative connections in the correlation matrix, setting the diagonal of the matrix to 0, and normalizing the matrix such that each column sums to 1. Therefore **R** contained only weighted (normalized) positive connections from the original correlation matrix. External signal, **E**, was only present at time t = 0, where the 50 seed nodes were set to 1, and all other nodes were 0. The seed nodes for the external signal were randomly selected from the population of nodes that were not deleted. The equation was iterated for 100 time steps. This spreading activation model was tested on the original network and the networks with nodes removed, where 5% through 80% of the nodes were removed in increments of 5%. By examining the total activation in the system over the course of the simulation, we evaluated the impact of removal of highly central nodes on the ability of information to spread through the network. Here, total activation is defined to be the sum of activity values across all nodes in the network at a given time during the simulation. This procedure was performed on 5 subjects. Additionally, the impact of targeting low degree nodes was examined in a single subject in order to further investigate the findings in [16], where the targeted removal of low degree nodes had a greater impact on the dynamics of a network containing coupled oscillators than high degree nodes. For this experiment, we removed nodes that were the top 5% through 30% highest centrality nodes as well as the 5% through 30% lowest centrality nodes, in increments of 5%. Seed nodes were again randomly selected from the pool of remaining nodes in the networks.

Varying the ratio α/γ results in a phase change in the spreading activation model. When α/γ is small, the total activation in the system decays to zero over time (referred to as Phase I), but as α/γ increases, the system enters a regime where the activation builds exponentially in a small component of the system, referred to as Phase II [26]. We chose α = 1 and tuned gamma until the original networks exhibited Phase II behavior, resulting in α/γ = 0.96.

Changes in dynamics were also evaluated by embedding a coarser form of each network into an agent-based model called the agent-based brain-inspired model (ABBM) described in [27]. An agent-based model is a collection of agents that interact with one another by following simple rules. The rules used here were inspired by the work of Stephen Wolfram [28], who has been a major contributor to the study of cellular automata. In this case, agents are represented by the nodes of the functional brain network, and links in the network represent communication pathways between agents. Each agent possesses a state, which can be either on or off, and may update its state based on the states of all connected neighbors by following one of Wolfram's Rules. Due to the computational demand of this model, these networks were constructed by parcellating the brain volume of each subject into 90 anatomical regions using the AAL (automated anatomic labeling) atlas [29]. The time series of all voxels belonging to a particular ROI were averaged in order to create 90 ROI time series. These time series were cross-correlated to construct a 90×90 ROI correlation matrix containing positive and negative connection weights. A threshold was applied to these networks to preserve only strong positive or negative connections while preventing fragmentation. Therefore, positive and negative weighted links were present in the ROI networks. The process of creating the ROI networks and the mechanisms underlying the ABBM are described in full in a prior publication [27].

These ROI networks were selectively attacked by removing 10% of the nodes (9 regions) with the highest centrality, at random, or with the lowest centrality. Slight modifications to the centrality metrics were necessary in order to calculate these metrics in the weighted, signed correlation matrix. Degree was calculated as the sum of the absolute value of the weights of all links belonging to a node. Leverage and eigenvector centrality, which depend only on the degree of the node and its connected neighbors, were calculated using this definition of degree. The weighted form of betweenness was calculated on the absolute value of the correlation matrix using the MATLAB BGL package (http://dgleich.github.com/matlab-bgl/).

The impact on dynamics was evaluated by testing the ability of the attacked agent-based model to solve the density classification problem, a problem originally utilized to evaluate whether a one-dimensional cellular automaton (CA) could support computation [30]. A CA can be thought of as belonging to a class of agent-based models, where agents are spatially embedded as adjacent cells. The goal of the density classification problem is to find a rule that can determine whether greater than half of the cells in a CA are initially in the on state. If the majority of cells are on (i.e. density >50%), then by the final iteration of the CA, all cells should be in the on state. Otherwise, all cells should be turned off. The system should be able to do this from any random initial configuration of node states. The key is that each node receives input from only a few other nodes in the network. Each node must decide based on this limited information whether to turn on or off in the next time step, resulting in network-wide cooperation without the luxury of network-wide communication. The rule and model parameters that must be used in order to perform this task are identified using a search optimization technique known as genetic algorithms. We have demonstrated that the ABBM is able to perform the density classification task with a high level of accuracy across a range of densities, while null models with randomized connectivity are not successful, indicating that the topology of the brain network is amenable to computation. Here we wished to determine how targeted removal of high centrality nodes would impact performance on this task.

**Table 1** contains a summary of treatments of the functional brain networks used in each procedure for evaluating network structure and dynamics.

**Table 1:** Summary of networks used to evaluate network topology and dynamics.

	Topology			Dynamics	
	S	Eglob	Eloc	SA	ABBM
Number of nodes	15996	15996	15996	15996	90
Threshold?	Yes	Yes	Yes	No	Yes
Weighting type	Binary	Binary	Binary	Weighted (+)	Weighted (+, −)

S: size of giant component, E_glob_: global efficiency, E_loc_: local efficiency, SA: spreading activation, ABBM: agent-based brain-inspired model.

## Results

### Topological analyses

**Figure 2:**
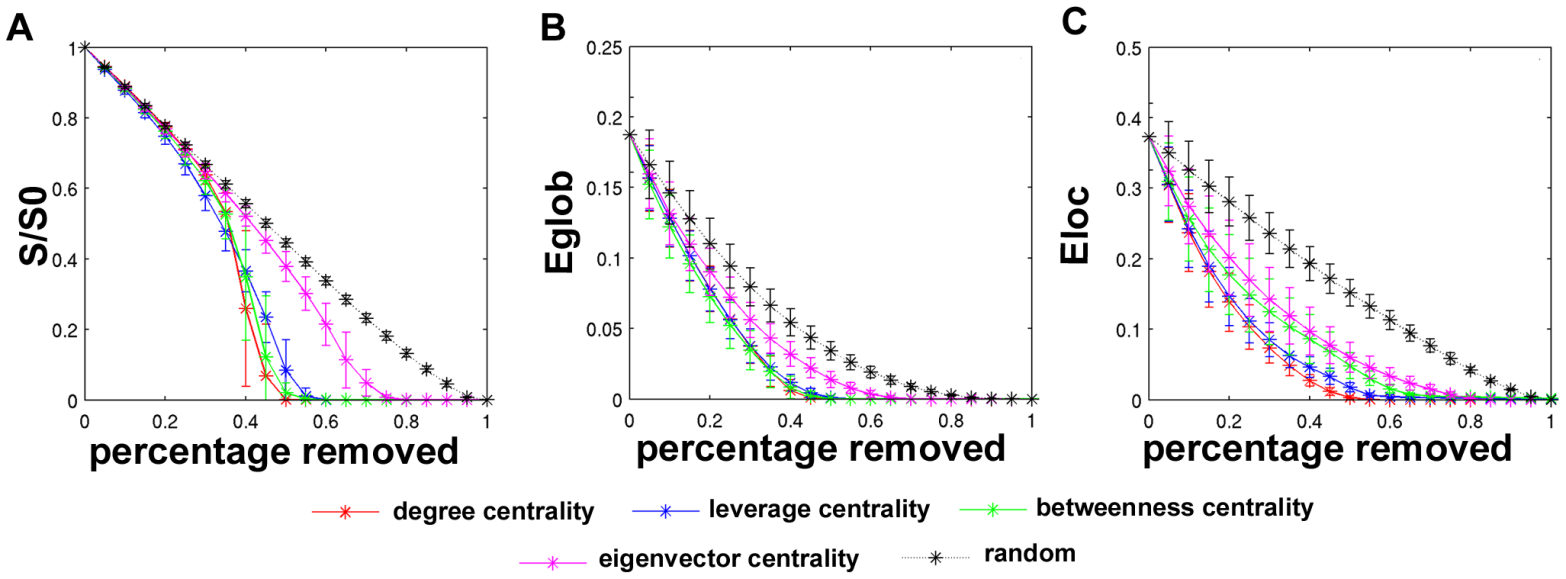
Topological changes in brain networks due to targeted attack and random failure. Panels depict changes in the size of the giant component (A), global efficiency (B), and local efficiency (C). The size of the giant component (S), was normalized to its original size (S0) in order to provide a consistent upper bound across subjects. All curves represent averages across 5 subjects, and error bars indicate standard deviations. Four centrality metrics were used to identify hubs: degree centrality (red), leverage centrality (blue), betweenness centrality (green), and eigenvector centrality (pink). Random failure (black) is included for comparison.

The quantities used to analyze the topological changes to the functional brain networks were the size of the giant component, global efficiency, and local efficiency. Each time the highest centrality nodes were identified and eliminated from the network, these three measures were recalculated and plotted along a curve. **Figure 2** contains these curves, averaged across the networks of 5 subjects. The size of the giant component, *S*, was normalized to the size of the giant component of the original network (*S0*). As the network nodes were selectively removed, the size of the giant component decreased, but did not show a dramatic reduction until nearly 40% of the nodes were eliminated from the network, regardless of the type of centrality used to identify hubs (**Figure 2**). Eliminating these hubs steadily decreased the global and local efficiency of the network as well. When comparing the removal of nodes based on different centrality metrics, removing nodes with high eigenvector centrality had the least effect on the networks. Network metrics declined visibly less for the removal of high eigenvector centrality nodes compared to degree or leverage, when evaluating all three of the network metrics. Targeted attack on high betweenness nodes was not highly different from degree or leverage, but it is notable that betweenness was also not highly different from eigenvector centrality when assessing local efficiency. **Table S1, Table S2, and Table S3** in **Text S1** show the ranges where there was a statistically significant difference in the size of the giant component, global efficiency, or local efficiency depending on the type of attack.

Targeted attack and random failure were also evaluated on a network with randomized connectivity. This network was generated using the method described in [31], where a functional brain network was rewired such that the degree distribution was preserved. The size of the giant component, local efficiency, and global efficiency underwent noticeably steeper declines after targeted attack than the original brain networks. Results from this experiment can be found in **Text S2**.

### Dynamical analyses using a spreading activation model

**Figure 3:**
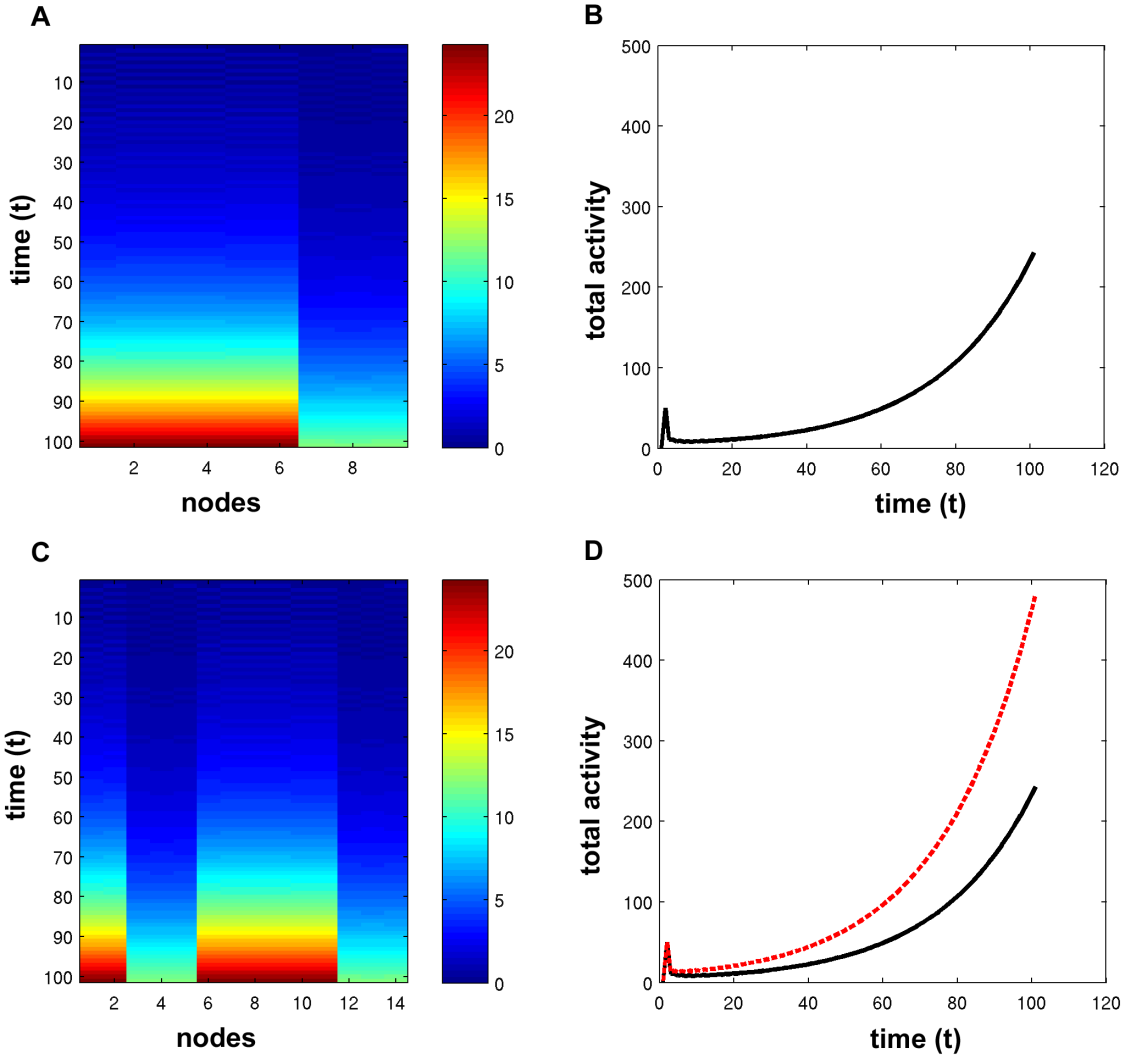
Spreading activation in an intact brain network and after targeted attack. (A) The activity of the 9 nodes shown exponentially increases, while the activity in all other nodes has decayed to zero. (B) The total activity, defined to be the sum of activation across all nodes at a given time step, illustrates the exponentially increasing activation. This network is exhibiting Phase II behavior. (C) After removing 20% of the highest degree centrality nodes, there are 14 nodes exhibiting exponentially increasing behavior, where nodes 6 through 14 are the same as pictured in (A). (D) The total activity of the attacked network (dashed red line) is greater than that of the intact network (solid black line).

Simulations using a spreading activation model were employed to demonstrate changes in network dynamics after targeted attack or random failure. **Figure 3** contains the results of the spreading activation model using the original (intact) network of one subject, as well as after attacking 20% of the highest degree centrality nodes. The activity within each node was computed at each time step, and during the simulation both the intact network and the attacked network were exhibiting Phase II activity. Recall that the Phase II activity pattern is characterized by a few nodes having activation that is exponentially increasing over time, while all other nodes in the network have activation that decays rapidly to zero. In the case of the original network, 9 nodes were exhibiting exponentially increasing activity. Panel A contains the two-dimensional color map of the time-series for the 9 nodes with exponentially increasing activity. The total activity in the network over the course of the simulation, defined to be the sum of activation across nodes at a given time step, is plotted in panel B. After the network was attacked, the number of nodes with exponential activity increased to 14, as pictured in panel C. The increase in nodes with building activity caused the total activity in the network, shown in panel D, to increase relative to the intact network.

**Figure 4:**
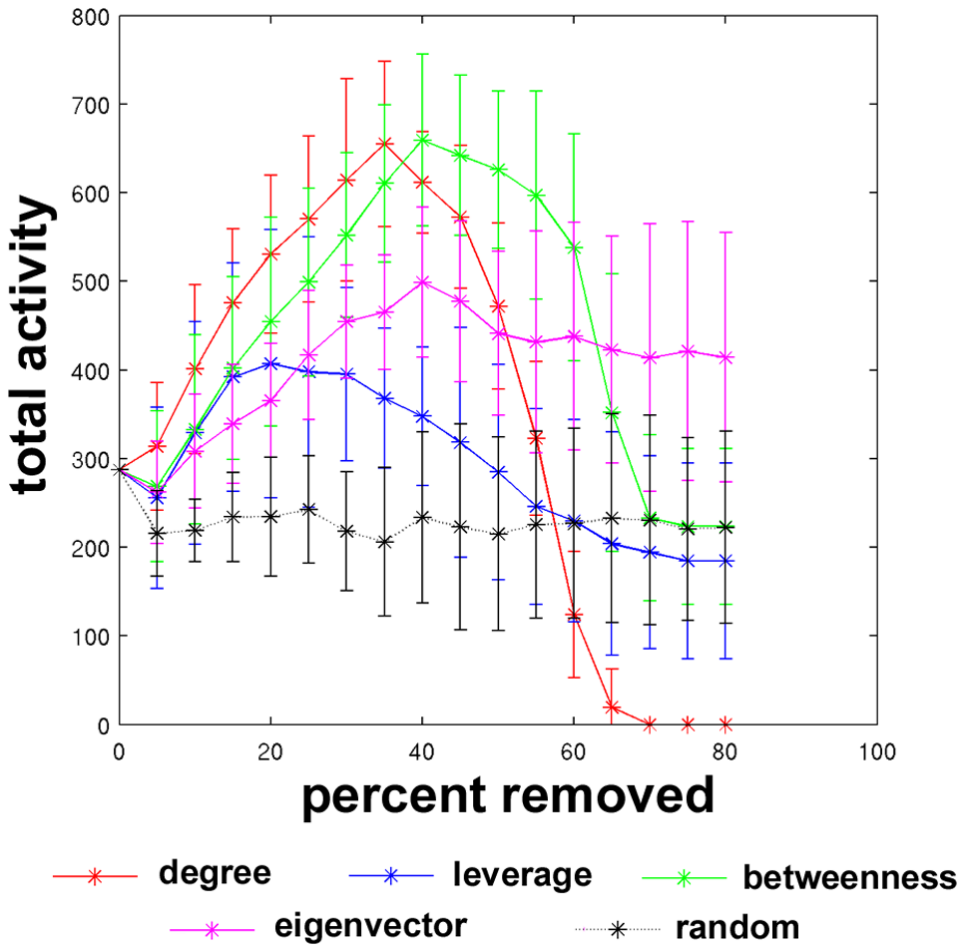
Changes in final total activity in networks as nodes are removed. The total activity attained at the end of the simulation (t = 100 iterations) is shown, averaged across all nodes in the network.

**Figure 4** shows the total activity achieved at the end of the simulation (t = 100), depending on the percentage of nodes removed using the four centrality metrics and for random failure. The original total activity is included, shown at 0% removed. These curves illustrate that the total activity achieved in the network increased depending on the extent of attack when high centrality nodes are targeted. Total activity was maximal when high degree and betweenness nodes were removed. Total activity actually decreased after random failure but removing further nodes had little effect beyond 5%.

All targeted attack curves in Figure 4 show a peak in total activity at a certain percentage of nodes removed. The peak in the curves corresponding to removal of high degree nodes occurs at 35%, and the peak corresponding to removal of high betweenness and eigenvector centrality nodes occurs at 40%. However, the curve corresponding to removal of high leverage nodes occurs much sooner, after 20% of nodes have been removed. According to Equation 1, the signal in a node is the sum of any external signal, previous activity that has not yet decayed, and new activity received from neighbors. The first two factors are not directly impacted by network attacks, as the seed nodes used for initial external signal are held constant as attacks are performed, and the decay factor is not dependent on the connectivity matrix. However, the changes in network connectivity will impact the spread of activity from neighboring nodes. Leverage centrality is designed to identify nodes that are connected to more nodes than their neighbors, and therefore control the content and quality of the information received by their neighbors. Therefore, leverage centrality tends to identify hubs whose directly connected neighbors would be negatively impacted by the loss of those hubs. As high leverage centrality nodes are removed, the remaining nodes that are highly dependent on high leverage nodes are not receiving as much signal. Therefore, leverage has the largest effect on hindering the spread of activation as measured via the peak in the total activation curves.

**Figure 5:**
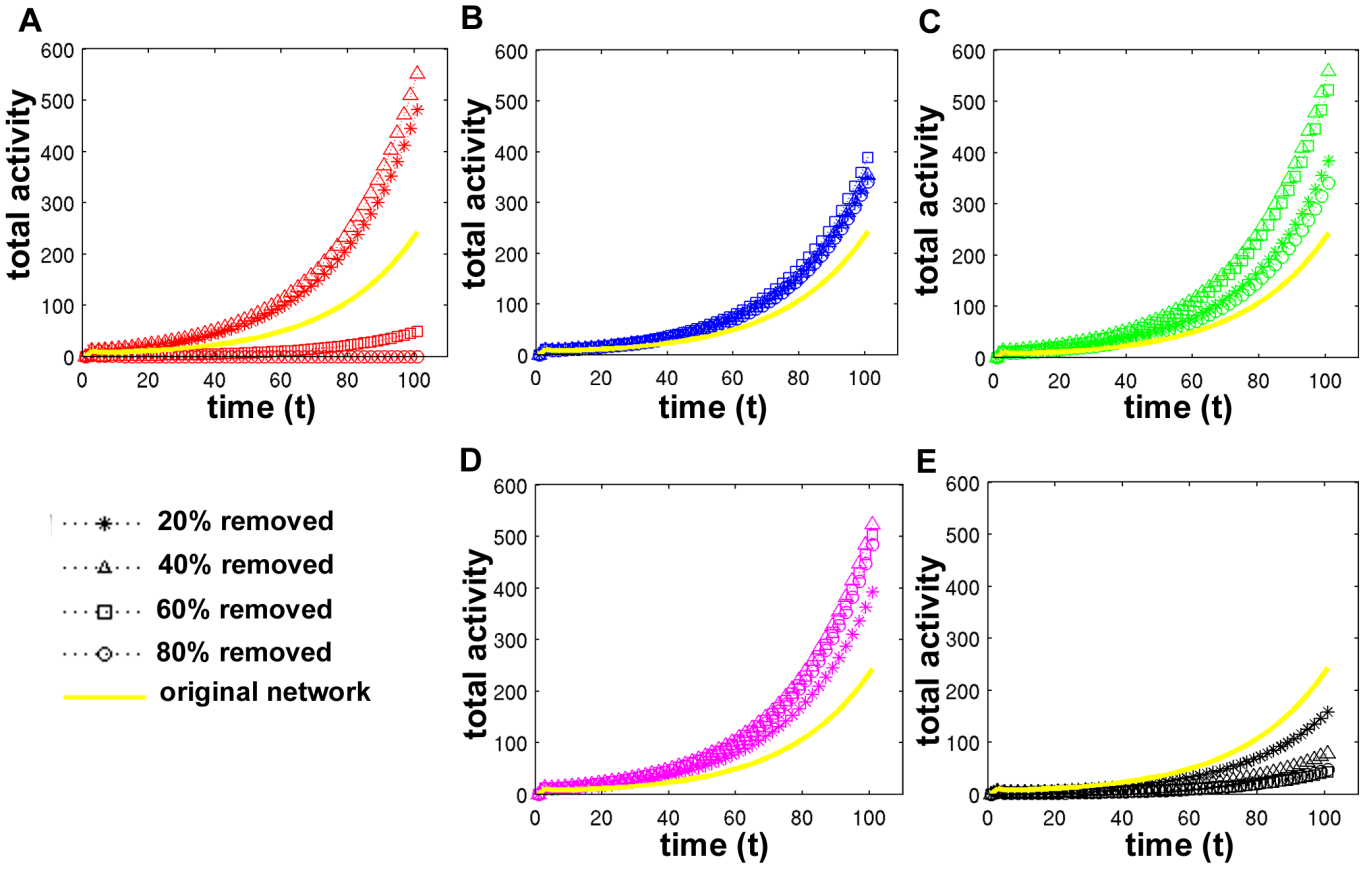
Total activity in the network after targeted attack and random failure in an example subject. Nodes are targeted by degree (A), leverage (B), betweenness (C), and eigenvector centrality (D), as well as at random (E). Curves represent networks after removing 20% (stars), 40% (triangles), 60% (squares), and 80% of the network (circles). The total activity of the original network is shown in yellow for comparison.

Despite the change in total activation, full activation curves demonstrated that, in the majority of cases, the networks remained in Phase II after targeted attack or random failure (**Figure 5**). The exceptions were networks where 70–80% of high degree centrality nodes were removed. These networks exhibited Phase I behavior, in which the total activity in the network decayed to zero. Activation curves are shown for the original network and after removing 20%, 40%, 60%, and 80% of nodes. While targeted attack of high centrality nodes generally increased the total activity in the network (to a point), random failure decreased the total activity for all levels of node removal. One-sample t-tests were performed to compare the final total activity across the 5 subjects after removing 20%, 40%, 60%, and 80% of the nodes. **Text S1** contains the resulting statistics.

**Figure 6:**
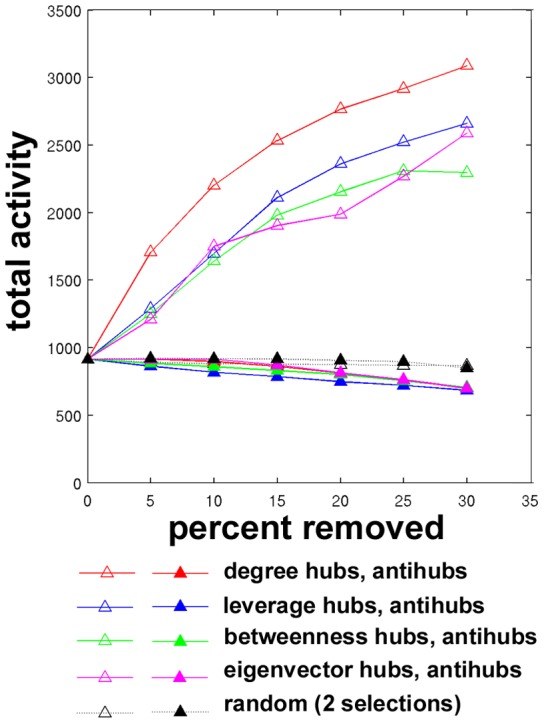
Changes in total activity after targeted attack or random failure. High centrality hubs (open symbols) and low centrality antihubs (filled symbols) were targeted for removal to compare the impact on dynamics in an example subject.

Total activity curves after removing high centrality hubs and low centrality antihubs are shown in **Figure 6** for a single subject. Recall that the seeds selected for this experiment were different from those used in previous experiments. Here, seed nodes were randomly chosen from the population of nodes not selected for removal as high centrality hubs or low centrality antihubs. It was necessary that seed nodes not be removed throughout the simulations in order keep the initial external signal, which originated at seed nodes, constant. As the seed nodes for this simulation were unique from the ones used previously (Figures 3–5), these networks achieved higher total activity values. As hubs were attacked, the total activation increased as in the previous simulation. In this case, the peak and subsequent decrease in final total activity are not captured, as in Figure 4, although the leverage curve peaked at 20% of nodes removed in the previous experiment. The profiles of the degree, leverage, betweenness, eigenvector, and random curves are similar to Figure 4. On the other hand, as antihubs were attacked, the activation decreased to a slightly greater extent than random.

Spreading activation experiments were also performed with seed nodes selected from the auditory cortex. However, the choice of seed nodes does not appear to change the observed dynamics in the spreading activation model. The results of this experiment can be found in **Text S3**.

### Dynamical analyses using an agent-based model

**Figure 7:**
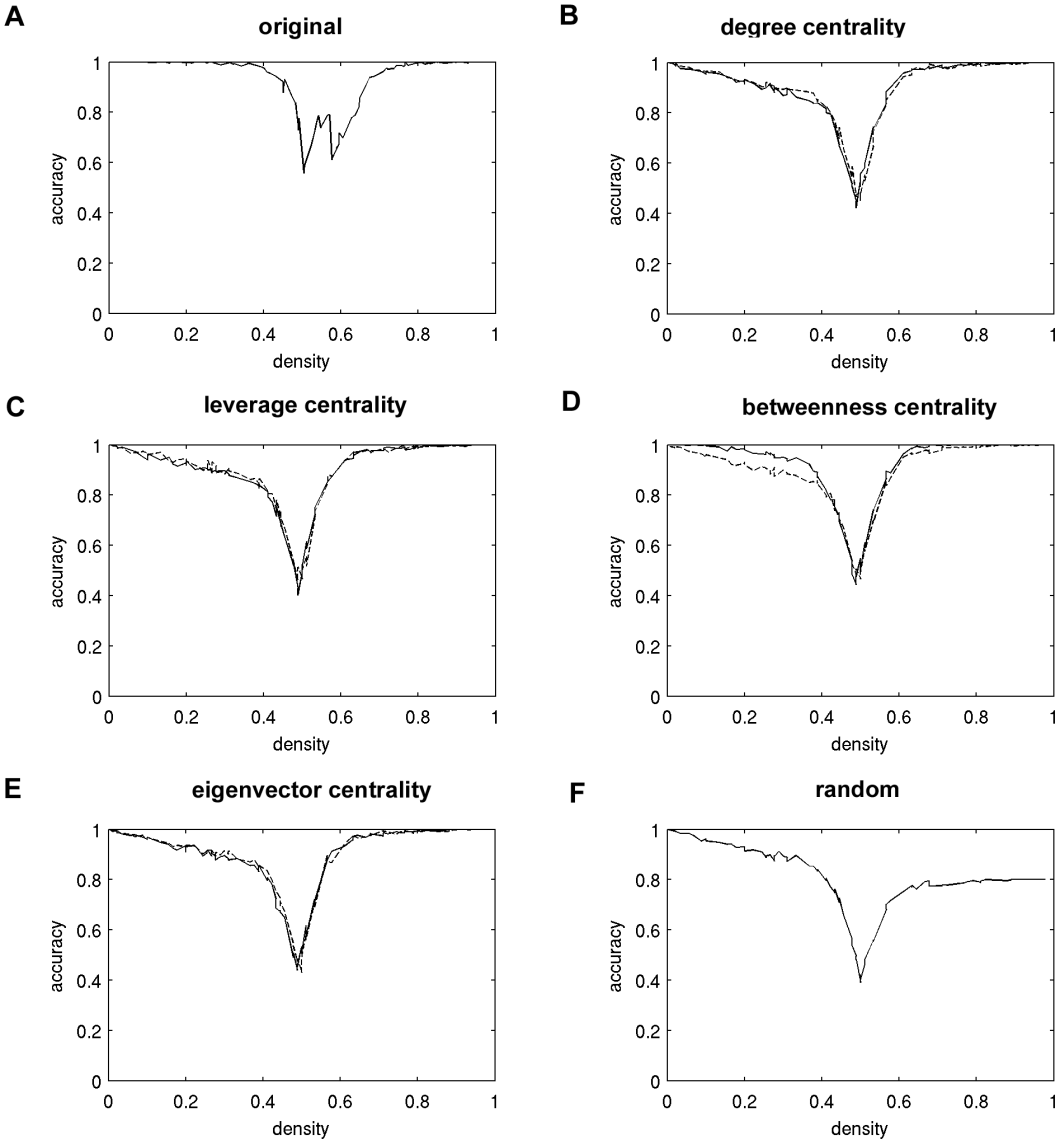
Impact of targeted attack on accuracy of solving the density classification task. Each panel depicts the mean accuracy curve for each type of targeted attack, averaged across subjects. The mean accuracy curves were generated for the original network (A) and after removing 10% of the nodes based on degree centrality (B), leverage centrality (C), betweenness centrality (D), eigenvector centrality (E), and at random (F). In panels B–E, solid lines indicate results when targeting hubs, and dashed lines indicate results when targeting antihubs.

In addition to the spreading activation model, simulations using an agent-based brain-inspired model (ABBM) were used to evaluate the impact of targeted attack and random failure on the ability of the ABBM to support global computation. A genetic algorithm was used to identify model parameters that enabled the ABBM to solve the density classification task using the original (intact) network (see Materials and Methods for details). The ABBM was then asked to solve the density classification task using the same parameters while operating on the networks with nodes removed. Accuracy curves were generated for each subject in order to evaluate the impact of targeted attack of hubs or antihubs and random failure on the ability of the model to solve this task. Mean accuracy curves, averaged across all subjects, are shown in **Figure 7**. On the left half of the density axis, where density <0.5, fewer than half of the nodes were on at the first time step. To the right, where density >0.5, greater than half of the nodes were initially on. All curves have a pronounced decrease in accuracy around density = 0.5, where the classification becomes more difficult. These accuracy curves show that, despite loss of highly central nodes, the ABBM maintains a high level of accuracy in solving the density classification task. This would suggest that the nodes that would be considered to be the most structurally integral components of the network have only marginal importance in information flow. On the other hand, the impact of random failure is greater than any type of targeted attack, specifically in higher density ranges. An ANOVA comparing mean accuracy across attack types revealed that targeting low centrality antihubs resulted in significantly decreased accuracy when compared to targeting hubs in only a select number of cases. Differences were found between leverage antihubs and eigenvector antihubs at density = 0.46 (mean difference 0.028, p = 0.019), leverage hubs and eigenvector antihubs at density = 0.53 (mean difference 0.064, p = 0.040), degree hubs and eigenvector antihubs at density = 0.61 (mean difference 0.046, p = 0.013), and leverage hubs and betweenness antihubs at density = 0.61 (mean difference = 0.058, p = 0.019). There were no significant differences in accuracy using the intact network versus any of the attacked networks.

## Discussion

We have presented a study on the topological and dynamical effects of targeted attack and random failure in human functional brain networks. Structural analyses employing local and global efficiency as well as the size of the giant component corroborate the findings presented in [3] in which the authors measured changes in the largest cluster size and the path length in functional brain networks, and further demonstrate that the choice of hub does not change the results appreciably. For any given centrality metric, nearly 40% of the nodes were removed before the size of the giant component qualitatively diverged from the random failure curve, which underwent a steady decrease as nodes were removed (although statistically significant differences exist much earlier). The reduction in local and global efficiency due to targeted attack followed curves only slightly steeper than random failure, with the effect on local efficiency somewhat greater than global efficiency. Global and local efficiency capture characteristics of the network structure that lend themselves to efficiency of information transfer. High local efficiency indicates topology that is conducive to local processing specificity, and topology with high global efficiency is amenable to long range information sharing. The topological characteristics that give the brain networks good local efficiency and reasonably high global efficiency are preserved, even when highly central nodes are targeted. Seemingly, whether high degree, betweenness, leverage, and eigenvector nodes are targeted, the result is the same: the topology of the functional brain network is relatively resilient to targeted attack.

Dynamical simulations using the spreading activation model revealed similar findings for information spreading across functional brain networks. Although targeted attack modified the total activity in the system at the end of the simulation, there was no phase transition in the overall behavior. The intact networks displayed Phase II activity, characterized by a limited number of nodes exhibiting exponentially increasing activity, while the activity in all other nodes decayed to zero. Random failure had very little impact on the total activity in the system. In contrast to random failure, the total activity in the network increased initially as high centrality nodes were targeted for removal, indicating that the signal was pooling in a number of nodes to a greater extent than before the attack, driven by the α parameter in the spreading activation model. Subsequently, as an increasing number of nodes were removed from the network, the final total activity decreased. Despite these quantitative changes, there was very little qualitative change in the system across all levels of targeted attack. It is important to note that the overall qualities of the system dynamics did not change. Despite initial expectations based on the work by Tanaka et al. mentioned previously, targeted removal of low centrality antihubs, while decreasing the final total activity, did not have a greater effect than targeted removal of highly central hubs. As Tanaka et al. note, low centrality nodes can maintain higher levels of activity because they do not spread their activity to many other nodes, while high centrality nodes tend to disperse activity to many other nodes. In the spreading activation model, when low centrality nodes are removed, less activity is allowed to pool, and the decaying term (γ) in the spreading activation model drives the behavior. On the other hand, removing high centrality nodes and their accompanying links decreases the dispersion of activity, and furthermore allows for increased pooling by simultaneously lowering the centrality of their former neighbors; therefore the total activity in the system increases.

The density classification task, rather than modeling the diffusion of information, tests whether a system can support computation. The agent-based brain-inspired model is constructed using the structure of the functional brain network. The agents in the model must make a collective decision (turn on or turn off) in order to solve the density classification task. As the network structure changes due to targeted attack or random failure, the information shared between nodes changes. Previously, we demonstrated that randomized connectivity patterns are not well suited to the density classification task, but that the functional brain network is. Therefore, we tested whether changes in network topology would impact the ability of the ABBM to make decisions. While targeted attack of hubs or antihubs impacted the accuracy to some degree, the average accuracy over a range of densities was still high, much higher than the accuracy of null models with randomized connectivity shown in [27]. Random failure resulted in a greater decrease in accuracy than targeted attack.

The density classification task is not a trivial problem. Each agent is supplied with a limited amount of local information, and must infer the state of the entire system. Furthermore, simply using the majority rule, where an agent chooses to take the state that the majority of its neighbors have taken, is not effective at solving this task [30]. Rather, the system must evolve a complex, yet simple, rule that can solve this task over just a few time steps, and moreover can accomplish this for any initial configuration. Simply solving this problem alone is notable, but solving it after 10% of the most central hubs and their accompanying links have been removed is an even more impressive feat. The fact that random networks with the same degree distribution as the brain network cannot solve this task would indicate that the network topology that enables the brain network to solve the density classification task remains intact after removal of central nodes. Since the global efficiency of the network remains high after targeted attack, one might be tempted to conclude that the efficient long range communication in the network lends it the ability to support computation. However, random networks, which cannot solve the density-classification task, are also characterized by high global efficiency. On the other hand, elementary cellular automata and other lattice-like networks with high local efficiency have been shown to be able to solve the density classification task with high accuracy [27], [30]. In these networks, nodes are clustered into well-connected groups and can share information readily, and therefore may be able to synchronize more easily. As high centrality nodes are targeted for removal from the voxel-wise functional brain networks, the networks maintain their high local efficiency to a much greater extent than randomized networks. In the agent-based model simulations, functional brain networks with 10% of the highest centrality nodes removed were still able to perform the density-classification task. These two findings together suggest that functional brain networks are able to perform computational tasks after targeted attack because the networks maintain their efficient local connectivity.

The two models we chose to employ for modeling dynamics on functional brain networks are the spreading activation (SA) model designed by Shrager et al. [26], and an agent-based brain-inspired model (ABBM), originally introduced in a prior publication [27]. The SA model and the ABBM simulate the flow of information in two disparate ways. We chose the SA model, a type of diffusion model, due to its application in physiologically relevant settings over the past several decades. Spreading activation has been used in artificial intelligence applications such as studying semantic networks, natural language processing, and information retrieval, as it was designed to be a model for memory associations and recall [32], [33], [34], [35].

While diffusion models are prevalent, agent-based modeling takes a somewhat different approach to simulating information flow. The ABBM is used to examine information sharing dynamics that can produce a collective behavior in the system. While the ABBM does not replicate the exact mechanisms of the brain, the method of agent-based modeling is well suited to producing emergent behaviors, which is almost certainly necessary to produce the most complex human behaviors. In the ABBM, each agent collects and integrates the information received from each of its neighbors, distills the information to a binary signal, and makes a decision on whether to fire based on that signal. Although the ABBM operates on a far coarser scale, this process mirrors action potential generation in a neuron.

Other widely used models include artificial neural networks, which consist of a set of nodes which take an input, operate on the input using mathematical functions, and produce an output. The networks are then trained to perform a particular task by allowing connections and mathematical operations to change. Neural networks are used in many pattern recognition applications, such as detecting seizures in EEG data [36], [37]. The distinction between the ABBM and neural network approaches to modeling brain functions is that the ABBM uses the network architecture determined from human functional brain imaging data, whereas the structure of neural networks is often determined by a set of features and desired outputs. By using functional brain network connectivity, the ABBM is generalized to solve different tasks without the need to re-train the network structure.

Alternatively, some researchers model cognitive functions using physical microcircuits. Neural microcircuits are used in applications such as the Blue Brain Project [38], where brain-like neural structures are modeled using a supercomputer dubbed Blue Gene. The computer consists of a network of 4,096 interconnected integrated circuits. The enormous computational power of Blue Gene enables the machine to solve cognitive problems using a brute force approach (e.g. analyzing the result of any possible move in a game of chess). Although the computational capability of Blue Gene is impressive, the advantage of using a combination of genetic algorithms and agent-based modeling is the elimination of the need to evaluate all possible outcomes, but instead search the solution space is a systematic way.

The field of network science provides a multitude of measures to capture the characteristics of complex systems, but, paradoxically, the complexity of these systems makes the task of understanding their underlying mechanisms quite challenging. The brain is intrinsically difficult to study. The measures and simulations presented here are surrogates for understanding the structural and dynamic changes that can occur in the brain. One limitation of these simulations is that they do not account for functional specialization of the various brain regions, where specific brain regions are thought to play key roles in specific functions. Certainly, many case studies in history have shown that damage to certain locations in the brain have unique effects due to functional specialization (e.g., the famous Phineas Gage [39]).

The simulations presented here also do not account for neuroplasticity, which enables the brain to remap cortical functionalities in response to sustained injuries. One study by Rubinov et al. examined the impact of random failure and targeted attack of high betweenness nodes in a synthetic neuronal network with neuroplasticity. They showed that allowing for the addition of new nodes through synaptogenesis, even at rates much slower than simulated neuronal death, was able to combat the impact on global and local efficiency [40]. Perhaps incorporating similar components of neuroplasticity into the models used in this work would enable an even greater demonstration of resilience.

Despite these limitations, this work progresses our understanding of the resilience of the human functional brain network. Based on the topological and dynamical simulations presented here, we conclude that the functional human brain network is highly resilient to targeted attack, both in terms of network structure and dynamics.

## Supporting Information

Text S1 **Statistical analyses for determining significant differences in topological changes to brain networks after targeted attack using varying centrality measures.** The curves in Figure 2 of the main text corresponding to the size of the giant component, global efficiency, and local efficiency exhibit similar contours for different targeted attack types. However, there are statistically significant differences between the curves at many levels of attack.
(DOCX)

Text S2 **Targeted attack and random failure experiments in a network with randomized topology.** The randomized network was generated by rewiring a functional brain network so as to preserve the original degree distribution while removing topological properties such as clustering and community structure. The random network exhibits more vulnerability to targeted attack than the brain networks presented in the main text.
(DOCX)

Text S3 **Spreading activation simulations in functional brain networks, where seed nodes were chosen to have physiological relevance for sensory input.** The choice of seed nodes does not appear to significantly affect the dynamical observations in the spreading activation model.
(DOCX)
