# Supplementary material for: The Human Functional Brain Network Demonstrates Structural and Dynamical Resilience to Targeted Attack
Source: PLoS Comput Biol. 2013 Jan 24;9(1):e1002885. doi: 10.1371/journal.pcbi.1002885 (PMC3554573; doi:10.1371/journal.pcbi.1002885)
Supplement: Text S1 — Statistical analyses for determining significant differences in topological changes to brain networks after targeted attack using varying centrality measures. The curves in Figure 2 of the main text corresponding to the size of the giant component, global efficiency, and local efficiency exhibit similar contours for different targeted attack types. However, there are statistically significant differences between the curves at many levels of attack. (DOCX) [file pcbi.1002885.s001.docx]

**Text S1**

After performing targetted attacks and random failure experiments, the size of the giant component, global efficiency, and local efficiency of each network were recalculated. The data from these experiments are shown in Figure 2 of the main text. Following these experiments, a one-way repeated measures analysis of variance (ANOVA) was performed in order to determine where statistically significant differences in the curves exist. Table S1, Table S2, and Table S3 below list ranges (in 5% increments) where statistically significant differences were detected (p < 0.05).

While the size of the giant component curves for degree, leverage, and betweenness centrality follow very close contours, significant differences were present at many points along the curves. The leverage centrality curve was the first to significantly diverge from the random failure curve.

Global efficiency curves for all targetted attacks were significantly different from the random curve at every level of attack. The leverage global efficiency curve was not significantly different from the degree curve until 35% of hubs were removed, which is likely due to many high degree nodes also having high leverage.

Similarly, local efficiency curves for all types of targetted attack were significantly different from the random failure curve at all levels of attack. Different types of targetted attack were also significantly different from each other at the majority of points along the curves.

**Table S1. Range of percent of nodes removed where there was a significant difference in the size of the giant component between attack types (p<0.05).** Values in the table indicate percentage of nodes removed.

|  | Degree | Leverage | Betweenness | Eigenvector | Random |
| --- | --- | --- | --- | --- | --- |
| Degree | - | 5-35,45,65-95 | 5-10,40-45,55,65-95 | 5-15,35-70,80-85 | 35-95 |
| Leverage |  | - | 10-20,30-35,65-70 | 5-70,90-95 | 5-95 |
| Betweenness |  |  | - | 25-35,45-70,90-95 | 35,45-95 |
| Eigenvector |  |  |  | - | 35-95 |

**Table S2. Range of percent of nodes removed where there was a significant difference in global efficiency between attack types (p<0.05).** Values in the table indicate percentage of nodes removed.

|  | Degree | Leverage | Betweenness | Eigenvector | Random |
| --- | --- | --- | --- | --- | --- |
| Degree | - | 35-45,65-80,90 | 5-25,40,50-70,80,90 | 5-60,75-80 | 5-95 |
| Leverage |  | - | 5-45,60 | 5,15-60,75,90 | 5-95 |
| Betweenness |  |  | - | 5-60,75,90 | 5-95 |
| Eigenvector |  |  |  | - | 5-95 |

**Table S3. Range of percent of nodes removed where there was a significant difference in local efficiency between attack types (p<0.05).** Values in the table indicate percentage of nodes removed.

|  | Degree | Leverage | Betweenness | Eigenvector | Random |
| --- | --- | --- | --- | --- | --- |
| Degree | - | 15-95 | 5-95 | 5-75 | 5-95 |
| Leverage |  | - | 5-70,80-95 | 5-75,85-95 | 5-95 |
| Betweenness |  |  | - | 5-40,55-70,85-95 | 5-95 |
| Eigenvector |  |  |  | - | 5-95 |

Activation curves in the main text (**Figure 5**) demonstrated that, in the majority of cases, the networks remained in Phase II after targeted attack or random failure (the exceptions were networks where 70 – 80% of high degree centrality nodes were removed). One-sample t-statistic tests were performed to compare the final total activity across the 5 subjects after removing 20%, 40%, 60%, and 80% of the nodes. The mean total activity of the original networks was used as the null hypothesis mean. The total activity of the original networks was 218.6 +/- 48.9 (mean +/- standard deviation, arbitrary units). While the intact and targetted attack total activity curves in Figure 5 are qualitatively very similar, statistically significant differences exist at many attack levels. Differences due to random failure, on the other hand, were not found to be statistically significant. Table S4 contains the means and standard deviations of the final total activity after attacks.

**Table S4. Level of statistical significance in pairwise t-statistic comparison of mean final total activity in the intact networks and after removing 20%, 40%, 60%, and 80% of nodes.** * indicates statistical significance (p<0.05).

|  | 20% removed  (mean, std) | | 40% removed  (mean, std) | | 60% removed  (mean, std) | | 80% removed  (mean, std) | |
| --- | --- | --- | --- | --- | --- | --- | --- | --- |
| Degree | 530.5, 39.9 | * | 611.5, 57.1 | * | 123.8, 71.0 | * | 0, 0 | * |
| Leverage | 407.3,151.3 | * | 347.7, 78.2 | * | 229.8,114.1 |  | 184.5,110.7 |  |
| Betweenness | 454.4,118.1 | * | 659.1,97.0 | * | 538.1,128.1 | * | 223.4,88.2 |  |
| Eigenvector | 365.6, 64.2 | * | 498.9,84.9 | * | 437.9,128.5 | * | 414.0,140.7 | * |
| Random | 234.4,67.0 |  | 233.5, 96.7 |  | 226.7,107.1 |  | 222.4,108.1 |  |
