# Supplementary material for: The Human Functional Brain Network Demonstrates Structural and Dynamical Resilience to Targeted Attack
Source: PLoS Comput Biol. 2013 Jan 24;9(1):e1002885. doi: 10.1371/journal.pcbi.1002885 (PMC3554573; doi:10.1371/journal.pcbi.1002885)
Supplement: Text S2 — Targeted attack and random failure experiments in a network with randomized topology. The randomized network was generated by rewiring a functional brain network so as to preserve the original degree distribution while removing topological properties such as clustering and community structure. The random network exhibits more vulnerability to targeted attack than the brain networks presented in the main text. (DOCX) [file pcbi.1002885.s002.docx]

**Text S2**

Targeted attack and random failure experiments were performed on a network with randomized connections. This network was generated using a method whereby a functional brain network is rewired such that the degree distribution is preserved. This is performed by selecting two links at random and swapping the nodes at which the two links terminate. Since the two terminating nodes each lose a single connection, but gain a new connection, the degree of each node does not change. Each random rewire alters two links, and 1000 rewires were performed, where it was possible to select any given link to be rewired multiple times.

As was done for the original networks, nodes were selected for removing using degree, leverage, betweenness, and eigenvector centralities as well as at random. The network was attacked at 5% increments, until all nodes were removed. The size of the giant component, local efficiency, and global efficiency were measured after each attack.

**Figure S1** contains the resulting curves. Since random rewiring tends to introduce “short cut” links in the network, the initial global efficiency in this network was greater than the initial global efficiency in the original networks. Similarly, since random rewiring tends to reduce the clustered topology of brain networks, the initial local efficiency of the rewired network was lower than in the original networks. All three curves underwent noticeably steeper declines after targeted attack than the original brain networks, suggesting that the brain topology is more resilient to targeted attack than would be expected based on randomized topology. The impact of random failure on the randomized network is comparable to the original human brain networks.


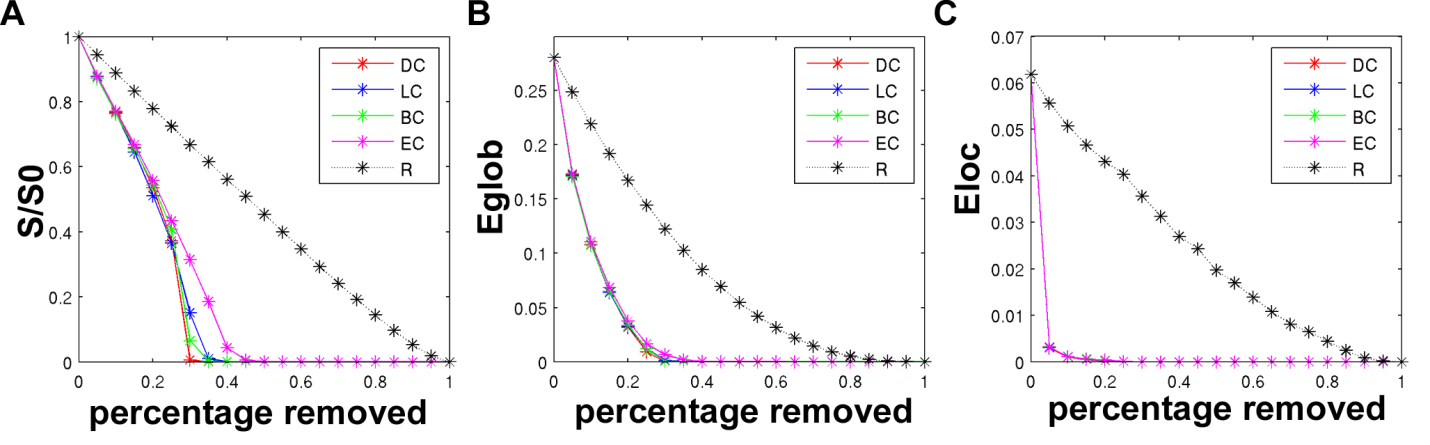


**Figure S1. Changes in the size of the giant component (A), global efficiency (B), and local efficiency (C) due to targeted attack and random failure.** When compared to the original brain networks (main text, Figure 2), this randomized network is more susceptible to targeted attack.
