# Supplementary material for: The Human Functional Brain Network Demonstrates Structural and Dynamical Resilience to Targeted Attack
Source: PLoS Comput Biol. 2013 Jan 24;9(1):e1002885. doi: 10.1371/journal.pcbi.1002885 (PMC3554573; doi:10.1371/journal.pcbi.1002885)
Supplement: Text S3 — Spreading activation simulations in functional brain networks, where seed nodes were chosen to have physiological relevance for sensory input. The choice of seed nodes does not appear to significantly affect the dynamical observations in the spreading activation model. (DOCX) [file pcbi.1002885.s003.docx]

**Text S3**

Here we present results from spreading activation simulations in functional brain networks, where seed nodes were chosen to have physiological relevance for sensory input. In this case, fifty seed nodes were selected from the left and right Heschl’s gyrus and superior temporal gyrus, as segmented by the Automated Anatomical Labeling Atlas. Seed nodes were selected to be 50 of the nodes in these regions which remained in the network when up to 60 percent of the nodes were removed. We attempted to maintain spatial clustering by selecting the first 50 nodes encountered from superior to inferior slices. Due to the restriction of choosing nodes from these four regions only, it was not possible to choose the same seed nodes for all subjects.

Note that the nodes removed at each attack level are the same nodes removed in the simulations in the main text. The spreading and decay parameters were also held constant (α = 1, α/γ = 0.96), and the simulation was iterated for the same length of time (100 time steps). **Figure S1** depicts the location of seed nodes in brain-space. Similar to the experiments in the main text, we show the total activity in the network at the final time step after varying percentages of nodes have been removed (**Figure S2**), as well as total activity curves over the course of the simulation for the original network and after 20%, 40%, and 60% of the nodes were removed (**Figure S3**).

Because the seed nodes used in this experiment are different from simulations in the main text, the initial total activity, and the total activity after attacks, is slightly elevated. The total activity curves using auditory cortex seed nodes in Panel A of Figure S2 achieve higher peak total activity values than the curves using the original seed nodes in Panel B, but follow similar profiles. Additionally, the points at which each of the curves corresponding to targetted attacks reach their respective maxima in the same order for both simulations, although these peaks occur at greater attack levels.

The total activity curves shown in Figure S3 were generated using the same subject as the prior experiment with randomly chosen seed nodes (main text, Figure 5). Just as in the previous simulations, the total activity curves show that there is no phase change. A phase change may occur as more nodes are removed, as was observed after 70 - 80 percent of high degree nodes were removed in the previous experiments.

In summary, the choice of seed nodes does not appear to significantly change dynamical observations using the spreading activation model.


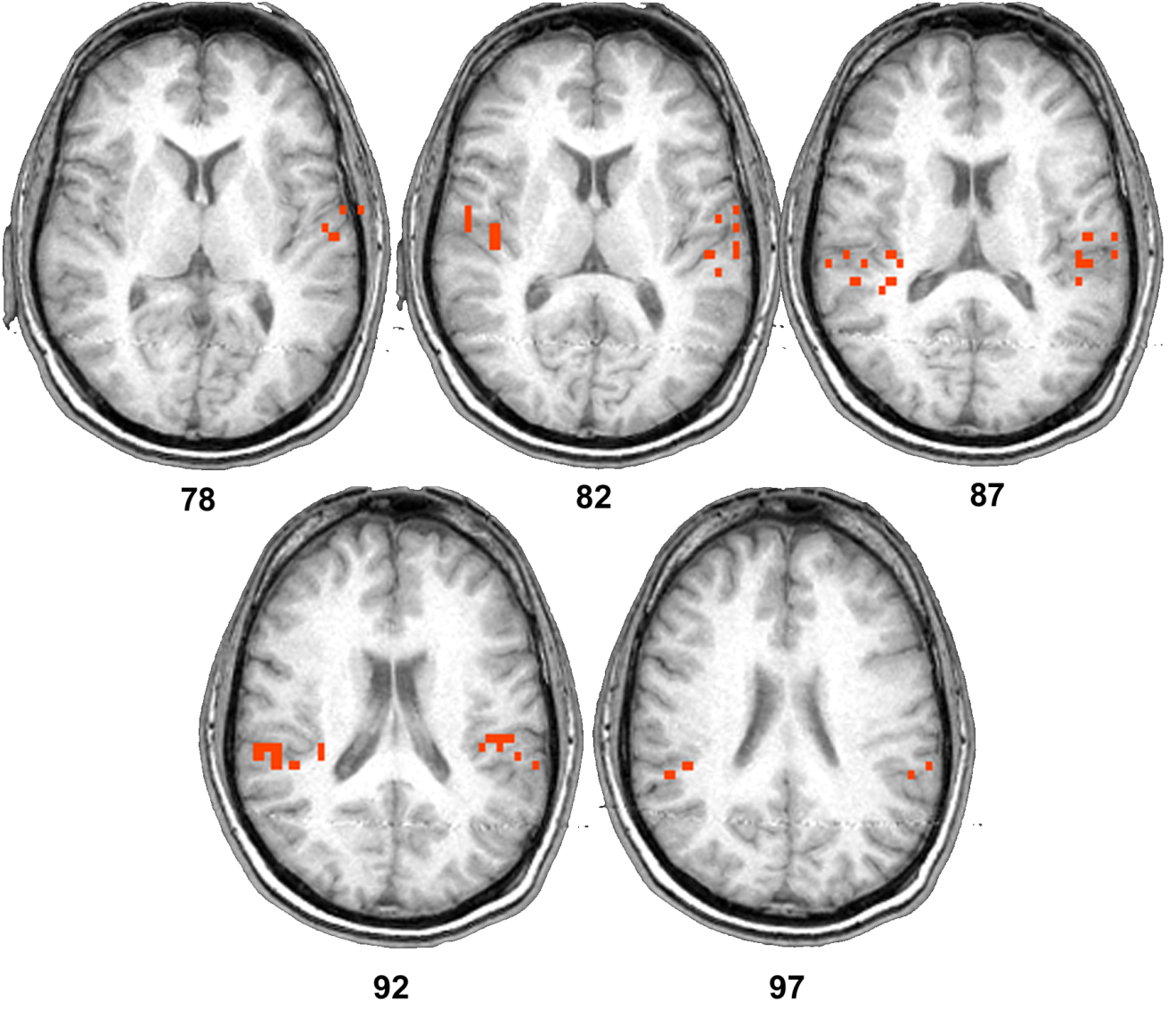


**Figure S1. Auditory cortex seed nodes used in spreading activation simulation.** A total of 50 seed nodes were selected.


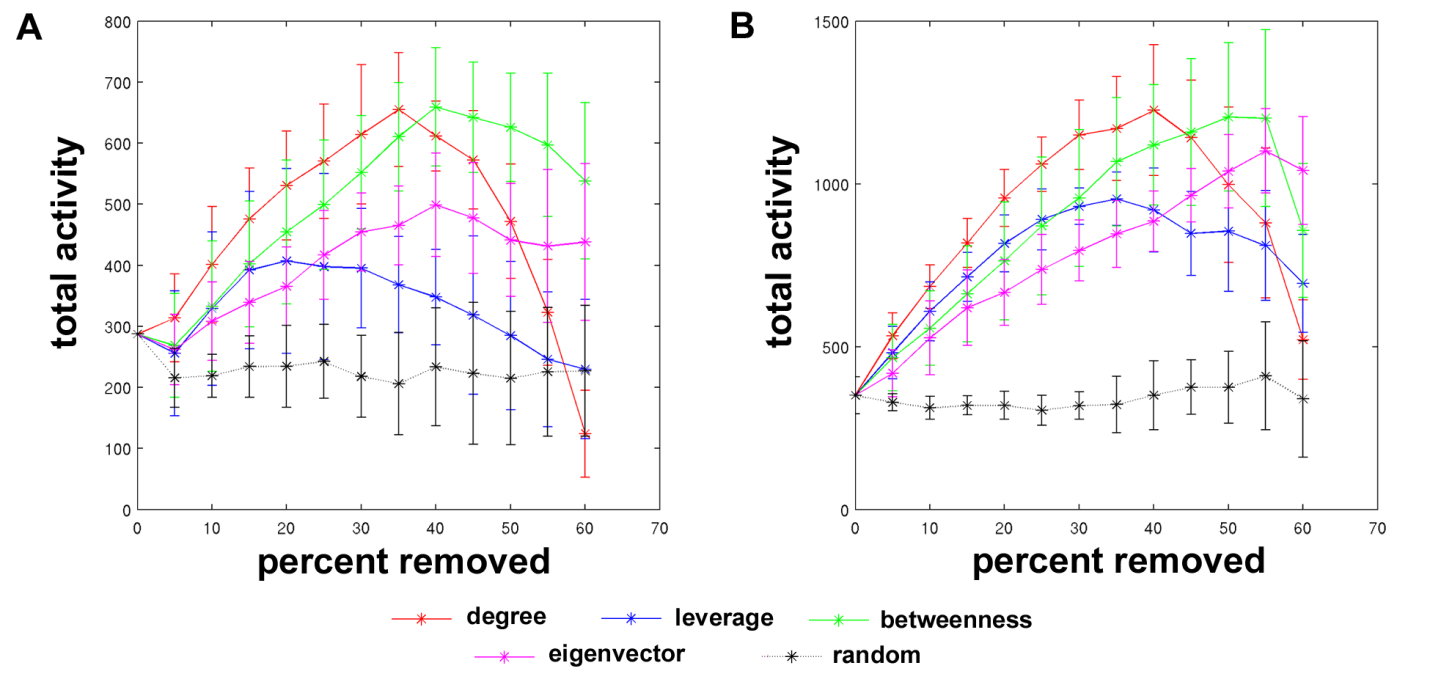


**Figure S2. Changes in final total activity in functional brain networks after attacks.** Panel A shows results using the randomly selected seed nodes used in the main text, and Panel B shows results using a selection of auditory cortex nodes as seed nodes**.**


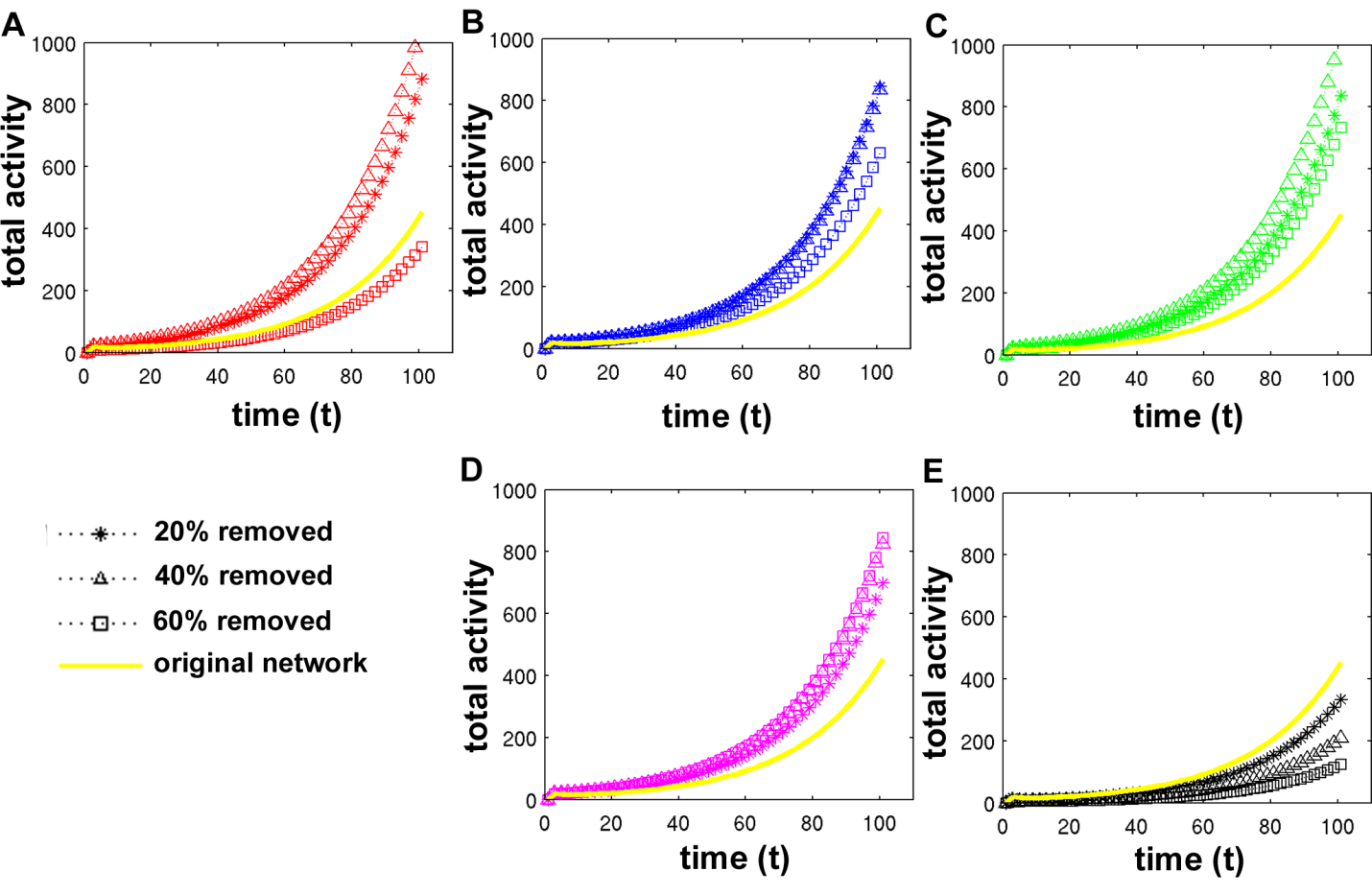


**Figure S3. Total activity in an example network after targeted attack and random failure, using a selection of auditory cortex nodes as seed nodes.** Nodes are targetted by degree (A), leverage (B), betweenness (C), and eigenvector centrality (D), as well as at random (E). Curves represent networks after removing 20% (stars), 40% (triangles), or 80% of the network (squares). The total activity of the original network is shown in yellow for comparison.
